# Aminophylline suppresses chronic renal failure progression by activating SIRT1/AMPK/mTOR-dependent autophagy: Suppression of chronic renal failure by aminophylline

**DOI:** 10.3724/abbs.2024049

**Published:** 2024-05-28

**Authors:** Xin Liao, Jieyi Lu, Zhifeng Huang, Jinai Lin, Miao Zhang, Huanru Chen, Xiaoqing Lin, Xia Gao, Sitang Gong

**Affiliations:** 1 Pediatric Department the First Affiliated Hospital of Jinan University Guangzhou 510030 China; 2 Department of Nephrology Guangzhou Women and Children’s Medical Center Guangzhou Medical University Guangzhou 510623 China; 3 Department of Burns and Wound Repair Surgery Guangdong Provincial People’s Hospital Guangdong Academy of Medical Sciences Guangzhou 510080 China

**Keywords:** chronic renal failure, aminophylline, SIRT1, mTOR, autophagy

## Abstract

Chronic renal failure (CRF) is a severe syndrome affecting the urinary system for which there are no effective therapeutics. In this study, we investigate the effects and mechanisms of aminophylline in preventing CRF development. A rat model of chronic renal failure is established by 5/6 nephrectomy. The levels of serum creatinine (SCR), urinary protein (UPR), and blood urea nitrogen (BUN) are detected by ELISA. Histological evaluations of renal tissues are performed by H&E, Masson staining, and PAS staining. Functional protein expression is detected by western blot analysis or immunofluorescence microscopy. Glomerular cell apoptosis is determined using the TUNEL method. Results show that Aminophylline significantly reduces the levels of SCR, UPR, and BUN in the CRF model rats. Histological analyses show that aminophylline effectively alleviates renal tissue injuries in CRF rats. The protein expression levels of nephrin, podocin, SIRT1, p-AMPK, and p-ULK1 are greatly increased, while p-mTOR protein expression is markedly decreased by aminophylline treatment. Additionally, the protein level of LC3B in CRF rats is significantly increased by aminophylline. Moreover, aminophylline alleviates apoptosis in the glomerular tissues of CRF rats. Furthermore, resveratrol promotes SIRT1, p-AMPK, and p-ULK1 protein expressions and reduces p-mTOR and LC3B protein expressions in CRF rats. Selisistat (a SIRT1 inhibitor) mitigates the changes in SIRT1, p-AMPK, p-ULK1, p-mTOR, and LC3B expressions induced by aminophylline. Finally, RAPA alleviates renal injury and apoptosis in CRF rats, and 3-MA eliminates the aminophylline-induced inhibition of renal injury and apoptosis in CRF rats. Aminophylline suppresses chronic renal failure progression by modulating the SIRT1/AMPK/mTOR-mediated autophagy process.

## Introduction

Chronic renal failure (CRF), also known as chronic kidney disease (CKD), is a severe urinary syndrome characterized by constant changes in kidney structure and a gradual loss of kidney function, usually resulting in hypertension, cardiovascular disease, edema, tumors, and even death [1]. Recent epidemiological studies have shown that CRF affects>7% of individuals worldwide. CRF is associated with multiple risk factors, including genetic mutations, acute and chronic kidney injuries, exposure to toxic reagents, a low nephron number at birth, obesity, and diabetes mellitus [2]. CRF development is known to be mediated by a loss of nephrons, remnant nephron hypertrophy, impaired glomerular filtration, and interstitial fibrosis [3]. The current clinical treatments for CRF include dialysis, kidney transplantation, and other replacement therapies. However, these forms of therapies are not available or affordable for all patients, especially for elderly patients with other complications [4]. New strategies to inhibit nephron loss or accelerate nephrogenesis and regeneration are urgently needed to prevent disease progression and improve the quality of life of CRF patients.

Aminophylline is usually a compound composed of theophylline and ethylenediamine. Theophylline is a caffeine analogue that acts as a nonselective inhibitor of phosphodiesterases, which catalyze the hydrolysis of cyclic nucleotides to modulate intracellular cAMP and cGMP levels. Because cAMP and cGMP are important second messengers associated with disease pathogenesis, aminophylline has long been used for the treatment of lung disorders such as asthma and chronic obstructive pulmonary disease (COPD). The therapeutic effects of aminophylline on lung disorders can be partially attributed to its ability to induce mitochondrial biogenesis and the resulting improvement in epithelial mitochondrial function. A previous study in a mouse model showed that aminophylline exerts a substantial anti-inflammatory effect due to its regulation of cytokine release, which might be useful for preventing inflammation-induced preterm parturition [5]. Furthermore, aminophylline has also shown promise for treating other human diseases, such as postdural puncture headache [6], hypercapnia [7], and irritable bowel syndrome [8]. More importantly, aminophylline demonstrated renoprotective effects in a mouse model of renal ischaemia‒reperfusion (I/R) injury, where it greatly improved renal function and inflammation indices [9]. Previous research has also shown that aminophylline can decrease serum creatinine level and prevent renal tissue damage in young patients with acute kidney injury (AKI) [10]. However, the possible use of aminophylline for repressing CRF development has not been studied.

Autophagy is an essential cellular process responsible for the degradation of cytoplasmic cargo, such as impaired organelles and proteins, by lysosomes. Autophagy is mediated by autophagy-related genes (ATGs), which are closely associated with the development of various human diseases such as diabetes, multiple sclerosis, neurodegenerative disorders, and cancers [11]. The induction of autophagy is tightly controlled by multiple cellular signaling pathways, including the sirtuin 1 (SIRT1), AMP-activated protein kinase (AMPK), and mammalian target of rapamycin (mTOR) pathways [12]. Moreover, autophagy plays dual roles in regulating cell apoptosis, and the induction of apoptosis can be suppressed by autophagy through repressing the activation of caspase proteins [13]. Furthermore, autophagy has been identified as a key cellular mechanism that modulates the structure, function, and homeostasis of kidney tissues and is thus widely involved in renal pathogenesis [14]. Knockout of the *ATG5* and *ATG7* genes promoted kidney dysfunction and the apoptosis of proximal tubular cells in a mouse model of AKI [15]. Importantly, alterations in the autophagy process were found to be associated with CRF and CKD pathogenesis [16], as well as with tubulointerstitial fibrosis and the AKI–CKD transition [17]. In addition, activation of autophagy was found to mediate the inhibition of CRF progression caused by O-GlcNAc transferase (OGT) or microRNA-376b depletion [18]. Hence, the potential regulation of autophagy by aminophylline in the context of CRF deserves exploration.

In this study, we investigated the possible inhibitory effects of aminophylline on CRF development and progression in a rat model and then explored the underlying mechanisms involving SIRT1-regulated autophagy and apoptosis. Our results provide new insights into the pharmaceutical functions of aminophylline and can serve as a basis for the use of aminophylline as a new therapeutic agent for CRF treatment.

## Materials and Methods

### Animal model establishment

A rat model of chronic renal failure was established by using the 5/6 nephrectomy method as previously described [19]. Briefly, the rats were first anesthetized by intraperitoneal injection of pentobarbital (50 mg/kg). Next, a single incision was made in the abdomen at a point 1.5 cm away from the left ribs to expose the left kidney, after which two-thirds of the left kidney tissue was removed. The incision was then closed with a silk ligature. One week later, the right kidney was removed by using similar surgical procedures. Rats in the sham group were also anesthetized and subjected to bilateral kidney exposure, after which the circumrenal adipose tissues were separated, and the incision was closed by suturing. The protocols for all animal experiments were approved by the Experimental Animal Usage Ethics Committee of Guangdong Medical Experimental Animal Center (Guangzhou, China).

### Animal grouping and treatment

To determine the efficacy of aminophylline, a total of 20 male Sprague-Dawley (SD) rats (200± 20 g) were allowed to adapt for one week in a specific pathogen-free environment and then randomly assigned to 4 different groups: a sham group ( *n*=5), a model group ( *n*=5), a low-dose aminophylline group ( *n*=5), and a high-dose aminophylline group ( *n*=5). Starting one week after nephrectomy surgery, aminophylline (high dose: 60 mg/kg; low dose: 20 mg/kg; HY-B0140; MCE, Monmouth Junction, USA) was given to the rats each day via intragastric administration [20] for a period of 4 weeks. Rats in the sham or model groups were given the same volume of distilled water.

To evaluate the mediating roles of SIRT1 signaling, a total of 25 SD rats were randomly assigned to a sham group, model group, model+resveratrol group (positive control; resveratrol is a drug used for clinical treatment of chronic kidney disease), model+aminophylline group, or model+aminophylline+selisistat group (selisistat is a potent and selective inhibitor of SIRT1). Aminophylline (60 mg/kg) was given to the rats via intragastric administration 2 h before surgery. Resveratrol (2 mg/kg; HY-16561; MCE) and selisistat (10 mg/kg; HY-15452; MCE) were administered to the rats via intraperitoneal injection 2 h before surgery [21].

To assess the involvement of autophagy, another group of 25 SD rats was randomly assigned to the sham group, model group, model+RAPA group, model+aminophylline group, or model+aminophylline+3-MA group. 3-MA is widely used as an inhibitor of autophagy because it inhibits class III PI3K. RAPA is an autophagy activator [22]. RAPA (2 mg/kg; S1039; Selleck, Houston, USA) or 3-MA (10 mg/kg; S2764; Selleck) was administered to rats via intraperitoneal injection 2 h before nephrectomy. Aminophylline (60 mg/kg) was given to the rats via intragastric administration 2 h before surgery.

### ELISA assays

After receiving their specified treatments, the rats were sacrificed, and blood and urine samples were collected and stored at ‒80°C for use in subsequent assays. The levels of serum creatinine (SCR), urinary protein (UPR), and blood urea nitrogen (BUN) were detected by enzyme-linked immunosorbent assay (ELISA) using commercial ELISA kits according to the manufacturer’s instructions. ELISA kits (#C011-2-1, #C035-2-1, and #C013-2-1) produced by the Nanjing Jiancheng Bioengineering Institute (Nanjing, China) were used to measure the levels of SCR, UPR, and BUN, respectively, in the model rats.

### Histological evaluations

Histological changes in rat kidney tissues were first evaluated by the standard hematoxylin-eosin (H&E) staining method. For Masson staining, samples of rat kidney tissue were first fixed in 10% formaldehyde and embedded in paraffin. Next, the paraffin-embedded sections were mounted onto slides and dewaxed, after which they were stained with hematoxylin for 5‒10 min at room temperature. Then, the sections were washed with running water, stained with Masson stain (0.7 g of ponceau, 0.3 g of acid fuchsin, 1 mL of glacial acetic acid, and 99 mL of distilled water) for 8 min at room temperature, treated with 1% phosphomolybdic acid, dehydrated, and finally observed under a microscope. Periodic acid-Schiff (PAS) staining of rat kidney tissues was performed using a commercial kit (ab150680; Abcam, Cambridge, UK) according to the manufacturer’s instructions. Briefly, slide-mounted tissues were hydrated, incubated in periodic acid solution for 4 min, immersed in Schiff’s solution for 12 min, rinsed with distilled water, and then incubated with Bluing reagent for 20 s. The slide-mounted tissues were then rinsed again with distilled water, dehydrated with graded alcohol solutions, cleared, and observed under a microscope. Renal pathology scores were used to assess the severity of kidney injury. For each kidney, 100 cortical tubules were analyzed from at least 5 different perspectives. A higher score indicated more severe damage (a maximum score of 10 per tubule). The scoring system was as follows: tubular epithelial cell flattening (1 point), brush margin loss (1 point), cell vesicular formation (1 or 2 points), cytoplasmic vacuolization (1 point), cell necrosis (1 or 2 points), interstitial edema (1 point), tubular lumen obstruction (1 or 2 points), and glomerular integrity (1 or 2 points).

### Western blot analysis

Total proteins were extracted from rat kidney tissues using a Total Protein Extraction kit (AMJ-KT0007; AmyJet Scientific, Wuhan, China) according to the manufacturer’s instructions. Next, a 25‒30 μg sample of rat kidney protein from each group was boiled in loading buffer at 100°C for 5 min and separated by 10%‒12% SDS-PAGE, and the protein bands were transferred onto PVDF membranes, which were subsequently blocked with 5% BSA solution for 2.5‒3 h at room temperature. Next, the membranes were incubated with diluted primary antibodies for either 2 h at room temperature or overnight at 4°C, washed 3 times with TBST, and then incubated with a secondary antibody diluted in TBST. Finally, the immunostained proteins were detected by using Pierce ECL western blot substrates (32106; Thermo Fisher Scientific, Waltham, USA) according to the manufacturer’s instructions. Glyceraldehyde-3-phosphate dehydrogenase (GAPDH) served as an internal standard. The antibodies used in the study were as follows: anti-Nephrin (1:4000, ab216341; Abcam), anti-Podocin (1:2000, ab181143; Abcam), anti-SIRT1 (1:2000, 8469; CST, Danvers, USA), anti-AMPK (1:2000, 5831; CST), anti-p-AMPK (1:3000, 50081; CST), anti-ULK1 (1:4000, 8054; CST), anti-p-ULK1 (1:2000, 14202; CST), anti-mTOR (1:2000, ab134903; Abcam), anti-p-mTOR (1:2000, ab109268; Abcam), anti-Beclin-1 (1:3000, ab207612; Abcam), anti-LC3B (1:4000, 2775; CST), anti-P62 (1:4000, ab109012; Abcam), anti-ATG5 (1:2000, ab108327; Abcam), anti-PARP (1:2000, 9532; CST), anti-cleaved PARP (1:3000, 9548; CST), anti-Caspase 3 (1:4000, 9662; CST), anti-cleaved Caspase 3 (1:2000, 9664; CST), and anti-GAPDH (1:5000, ab8245; Abcam), goat anti-rabbit IgG H&L (1:5000, ab6702; Abcam) and goat anti-mouse IgG H&L (1:5000, ab6708; Abcam).

### Immunofluorescence microscopy

An immunofluorescence kit (P0179; Beyotime, Shanghai, China) was used to detect LC3B expression in rat kidney tissues according to the manufacturer’s instructions. Briefly, sections of slide-mounted kidney tissue were dewaxed, rehydrated, subjected to antigen retrieval, and then fixed for 15 min at room temperature. Subsequently, the slides were incubated with blocking buffer for 1 h at room temperature with gentle shaking and then incubated with a primary antibody targeting LC3B (2775; CST). Next, the slides were incubated for 1 h in the dark with a fluorescent-conjugated secondary antibody [goat anti-rabbit IgG H&L (Alexa Fluor® 555); ab150078; Abcam], counterstained with DAPI, and finally observed under a fluorescence microscope (Nikon Eclipse C1; Nikon, Tokyo, Japan).

### TUNEL staining

Cell apoptosis in rat kidney tissues was detected using a one-step TUNEL cell apoptosis kit (C1088; Beyotime) according to the manufacturer’s instructions. Briefly, sections of slide-mounted rat kidney tissue were dewaxed with xylene, rehydrated, incubated with protease K solution for 30 min, washed three times with PBS, and then incubated for 1 h with 50 μL of TUNEL detection solution at 37°C in the dark. Next, the slides were washed again three times with PBS, counterstained with DAPI, and observed under a fluorescence microscope.

### Statistical analysis

Data are were analyzed using SPSS 20.0 software and presented as the mean±standard deviation. Differences between groups were evaluated by ANOVA (analysis of variance), and *P*<0.05 was considered to indicate statistical significance.

## Results

### Aminophylline suppressed chronic renal failure progression in rats

**Figure 1:**
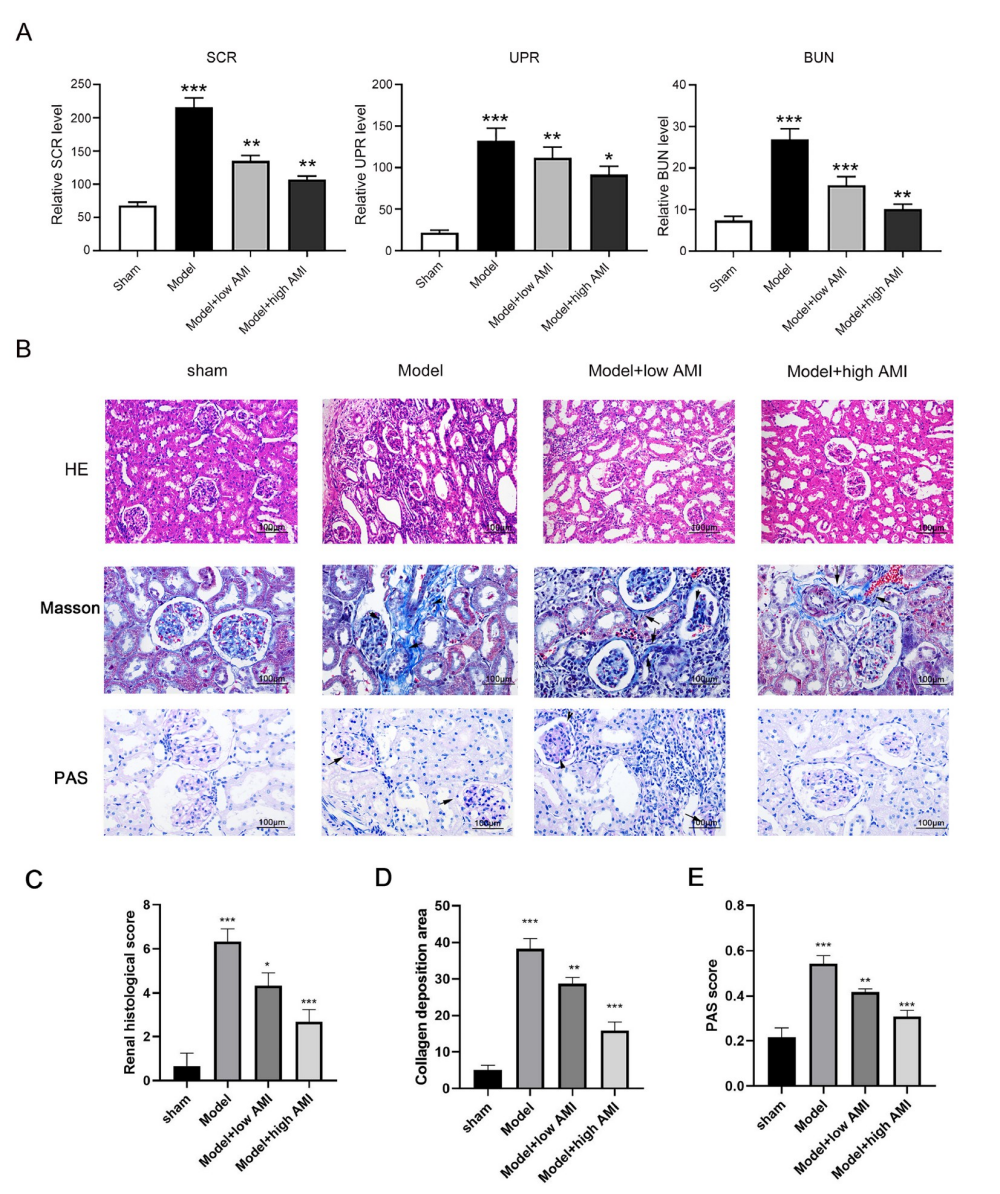
Figure 1 Inhibition of CRF progression by aminophylline treatment in the rat CRF model (A) The reductions in the SCR, UPR, and BUN levels in the CRF model rats caused by aminophylline treatment. Rats in the model groups were treated with a low dose (20 mg/kg) or high dose (60 mg/kg) of aminophylline, and their SCR, UPR, and BUN levels were detected by ELISA. (B) Alleviation of renal pathogenic changes, renal interstitial fibrosis, and extracellular matrix deposition in CRF model rats after treatment with aminophylline. Histological evaluations were performed by H&E, Masson, and PAS staining. (C) Renal histology scores determined by H&E staining. (D) Collagen deposition as shown by Masson staining. (E) PAS scores. The black arrows indicate areas of collagen deposition (Masson staining) and areas of glycogen deposition (PAS staining). AMI: aminophylline; SCR: serum creatinine; UPR: urinary protein; BUN: blood urea nitrogen; PAS: periodic acid-Schiff stain. * P<0.05, ** P<0.01, *** P<0.001 vs sham.

To investigate the therapeutic effects of aminophylline on CRF development, we first established a rat CRF model via 5/6 nephrectomy, as described in the Materials and Methods section. The levels of SCR (serum creatinine), UPR (urinary protein), and BUN (blood urea nitrogen) were detected to evaluate CRF progression in the model rats. Compared with those in the sham group, the levels of SCR, UPR and BUN in the CRF model group were significantly elevated but could be reduced in a dose-dependent manner by treatment with aminophylline ( Figure 1A). A high dose of aminophylline (60 mg/kg) produced greater decreases in the SCR, UPR, and BUN levels in the CRF model rats than a low dose of aminophylline (20 mg/kg) ( Figure 1A). H&E staining revealed significant congestion of the glomeruli, interstitial hyperplasia, inflammatory cell infiltration, and other pathogenic alterations in the model group, but these changes were also markedly alleviated by aminophylline treatment in a dose-dependent manner ( Figure 1B). Moreover, we analyzed our experimental results using a pathological scoring system. We found that aminophylline could reduce the scores and that a high dose of aminophylline had a stronger effect on the model rats ( Figure 1C). Masson staining showed that the model group also exhibited significant renal interstitial fibrosis, which was effectively suppressed by treatment with aminophylline ( Figure 1B,D). Finally, periodic acid-Schiff (PAS) staining revealed significant deposition of extracellular matrix in the glomerular mesangium of the model group, which was also substantially repressed by aminophylline treatment in a dose-dependent manner ( Figure 1B,E). These results indicated that aminophylline was capable of alleviating CRF progression in the model rats.

### Aminophylline modulated autophagy and repressed apoptosis in CRF model rats

**Figure 2:**
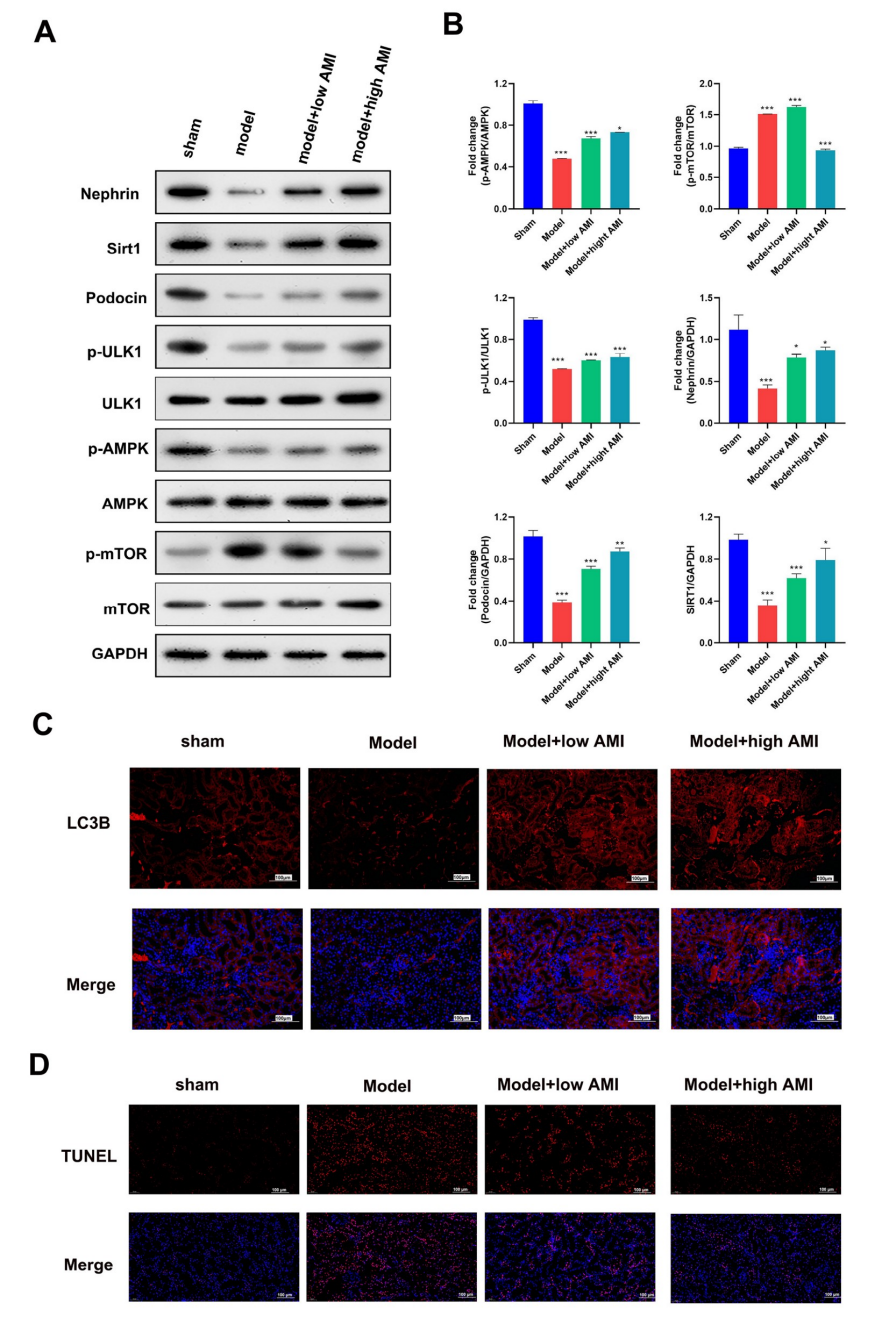
Figure 2 Aminophylline modulated autophagy and apoptosis in rat glomerular cells (A) Regulation of glomerular podocyte proteins and autophagy-associated proteins by aminophylline in CRF model rats. The relative protein levels in rat renal tissues were analyzed by western blot analysis, with GAPDH serving as an internal standard. (B) Quantitative analysis of SIRT1, podocin, p-AMPK, p-mTOR, p-ULK1, and nephrin protein expressions. (C) Changes in LC3B protein expression and distribution in CRF model rats treated with aminophylline. LC3B protein levels and distribution in cells were assessed by immunofluorescence staining. (D) Inhibition of glomerular cell apoptosis by aminophylline in CRF model rats. Rat renal cell apoptosis was detected by TUNEL staining. AMI: aminophylline; SIRT1: sirtuin 1; AMPK: AMP-activated protein kinase; ULK1: Unc-51-like kinase-1; mTOR: mammalian target of rapamycin; LC3B: microtubule-associated protein 1 light chain 3B. * P<0.05, *** P<0.001 vs sham.

To analyze the underlying cellular mechanisms involved, we examined the potential role of autophagy in regulating CRF progression by aminophylline treatment. Western blot analysis results showed that the levels of two glomerular podocyte proteins (nephrin and podocin), as well as the levels of SIRT1, phosphorylated AMPK, and autophagy-initiating kinase ULK1 (Unc-51-like kinase-1), were significantly lower in the model group than in the sham group ( Figure 2A,B). In contrast, the level of phosphorylated mTOR in the model group was significantly greater than that in the sham group. Importantly, we found that aminophylline treatment effectively abrogated the changes in the above protein markers in the model group. Immunofluorescence studies showed that the LC3B (microtubule-associated protein 1 light chain 3B) level in the model group, which was significantly lower than that in the sham group, and was also effectively increased by aminophylline treatment ( Figure 2C). In addition, TUNEL staining revealed that the increase in apoptosis of glomerular cells in the model group was also markedly repressed by aminophylline treatment ( Figure 2C). These results suggested that aminophylline modulated autophagy and inhibited the apoptosis of glomerular cells in CRF model rats.

### Aminophylline inhibited CRF progression in rats by regulating SIRT1 expression

**Figure 3:**
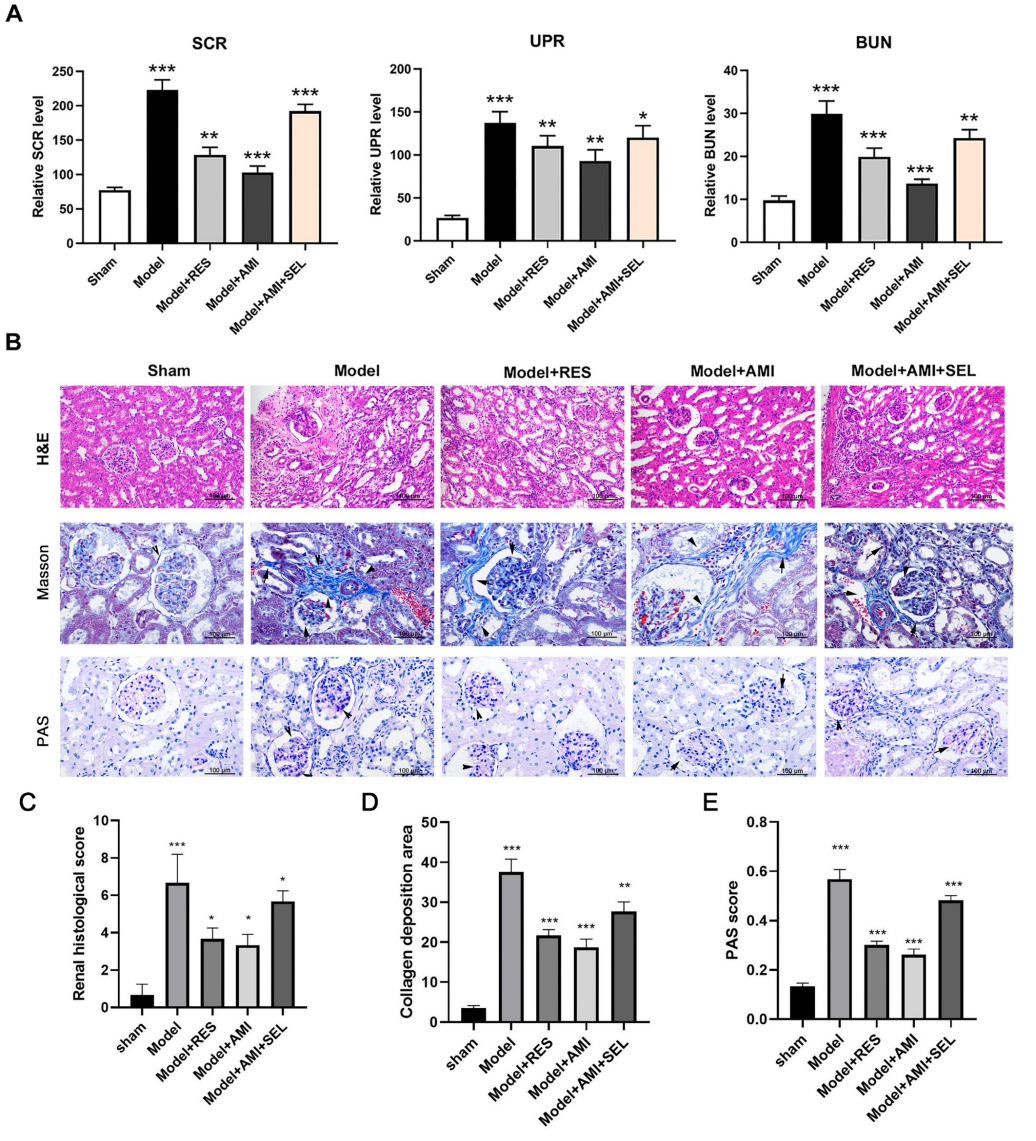
Figure 3 The mediating roles of SIRT1 in rat CRF inhibition by aminophylline (A) Changes in the SCR, UPR, and BUN levels in CRF model rats treated with resveratrol or a combination of aminophylline and selisistat. After continuous treatment for four weeks, ELISA was performed to measure the levels of SCR, UPR, and BUN in the rats. (B) Effect of resveratrol or selisistat treatment on the renal histological features of CRF model rats treated with aminophylline. Kidney tissue damage, interstitial fibrosis, and extracellular matrix accumulation in rat renal tissue were evaluated by H&E, Masson, and PAS staining, respectively. (C) Renal histology scores determined by H&E staining. (D) Collagen deposition as detected by Masson staining. (E) PAS scores. The black arrows indicate areas of collagen deposition (Masson staining) and areas of glycogen deposition (PAS staining). AMI: aminophylline; SCR: serum creatinine; UPR: urinary protein; BUN: blood urea nitrogen; RES: resveratrol; SEL: selisistat; PAS: periodic acid-Schiff stain. * P<0.05, ** P<0.01, *** P<0.001 vs sham.

To verify the role of SIRT1 in the aminophylline-mediated suppression of CRF development, we treated CRF model rats with the SIRT1 activator resveratrol or the SIRT1 inhibitor selisistat. Our results showed that the SIRT1 inhibitor resveratrol effectively reduced the levels of the SCR, UPR, and BUN in the CRF model rats ( Figure 3A). Furthermore, the reductions in the SCR, UPR, and BUN levels in the model rats caused by aminophylline were greatly attenuated by combined treatment with the SIRT1 inhibitor selisistat ( Figure 3A). Subsequent histological analyses revealed that renal tissue damage, such as glomerular congestion, interstitial hyperplasia, and inflammatory cell infiltration, in the model group was greatly alleviated by treatment with resveratrol ( Figure 3B). Furthermore, the inhibition of the above histological alterations in the model rats by aminophylline was partially abrogated by treatment with selisistat, and the pathology scores confirmed these findings ( Figure 3C). Similarly, resveratrol treatment suppressed the development of renal interstitial fibrosis in the CRF model group, and selisistat treatment partially mitigated the aminophylline-induced suppression of interstitial fibrosis in the CRF model group ( Figure 3B,D). In addition, the PAS results showed that treatment with resveratrol effectively inhibited the accumulation of extracellular matrix in the rat glomerular mesangium in the model group ( Figure 3B,E). Moreover, selisistat treatment substantially offset the effect of aminophylline on the suppression of extracellular matrix deposition in the glomerular mesangium regions of CRF model rats ( Figure 3B). These observations proved that SIRT1 activation mediated the inhibition of CRF progression caused by aminophylline treatment.

### Aminophylline modulated autophagy and apoptosis in CRF model rats by regulating SIRT1 signaling

**Figure 4:**
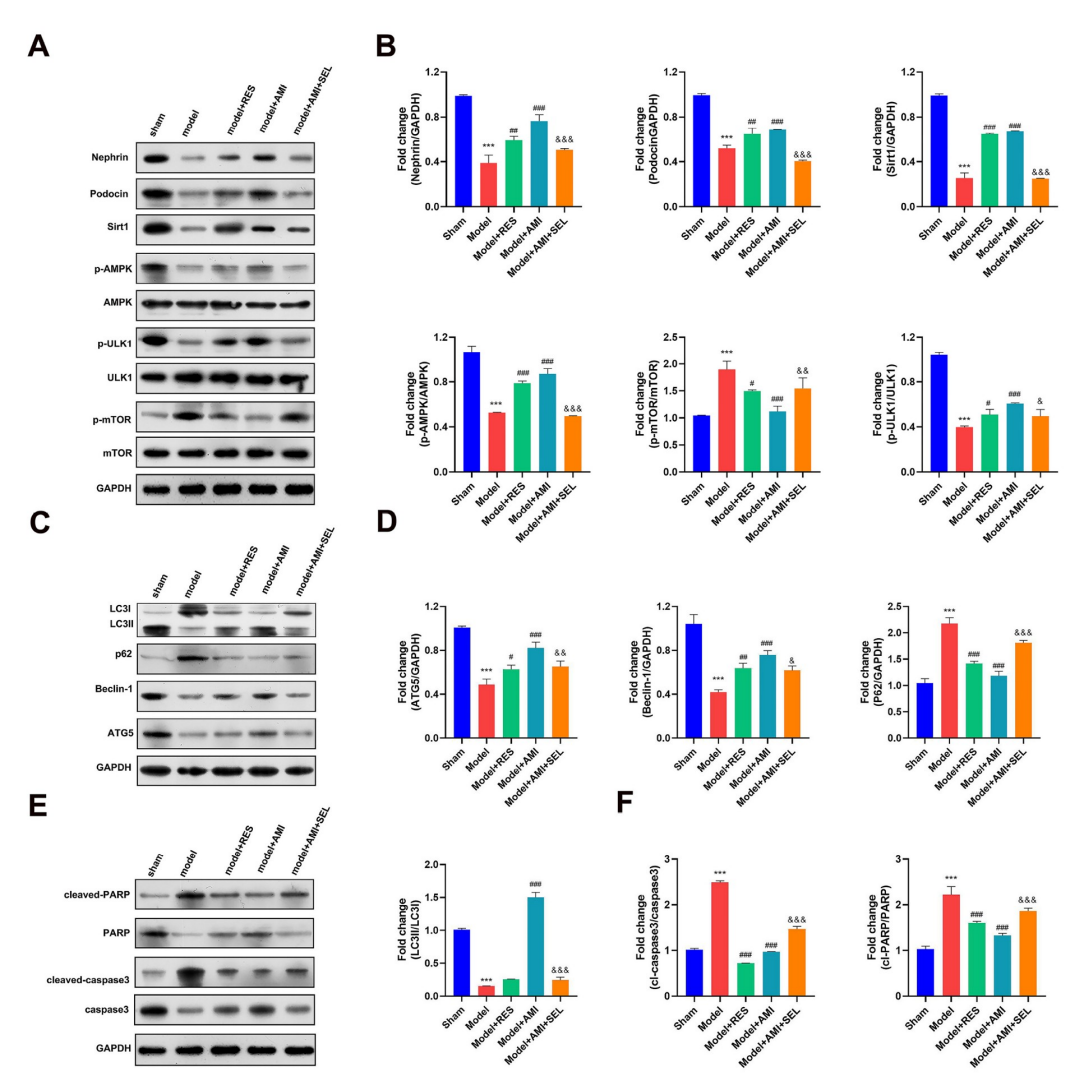
Figure 4 Roles of SIRT1 in aminophylline-regulated autophagy and its effects on apoptosis-related proteins in the CRF model (A) Changes in glomerular podocyte proteins and autophagy-regulating signaling proteins in CRF model rats treated with resveratrol or a combination of aminophylline and selisistat. Western blot analysis was performed to detect changes in protein levels in the CRF model rats, and GAPDH served as an internal standard. (B) Quantitative analysis of SIRT1, podocin, p-AMPK, p-mTOR, p-ULK1, and nephrin protein expressions. (C) The relative expressions of autophagy marker proteins in CRF model rats treated with resveratrol or a combination of aminophylline and selisistat. (D) Quantitative analysis of LC3B, Beclin1, p62, and atf5 protein expressions. (E) The influence of resveratrol or the combination of aminophylline and selisistat on apoptosis-related protein expression in CRF model rats. (F) Quantitative analysis of PARP and caspase 3 protein expressions. AMI: aminophylline; RES: resveratrol; SEL: selisistat; SIRT1: sirtuin 1; AMPK: AMP-activated protein kinase; ULK1: Unc-51-like kinase-1; mTOR: mammalian target of rapamycin; LC3B: microtubule-associated protein 1 light chain 3B; PARP: poly (ADP-ribose) polymerase. *** P<0.001 vs the sham group; # P<0.05, ## P<0.01, ### P<0.001 vs the model group; & P<0.05, && P<0.01, &&& P<0.001 vs the model+AMI group.

To gain additional insight into the cellular functions of SIRT1 during CRF inhibition by aminophylline, we detected changes in autophagy and apoptosis marker proteins in CRF model rats treated with a combination of aminophylline and selisistat. As shown in Figure 4, the levels of nephrin, podocin, SIRT1, and phosphorylated ULK1 in the renal tissues of the CRF model rats were greatly increased by resveratrol treatment ( Figure 4A,B). In contrast, the phosphorylation of the mTOR protein in CRF model rats was strongly repressed by resveratrol treatment. These changes in autophagy and apoptosis marker proteins were consistent with those observed in the model group treated with aminophylline alone and were significantly abrogated by the combination of aminophylline and selisistat. Moreover, in contrast to those in the sham group, the levels of the autophagy markers Beclin-1, LC3B-II, and ATG5 were markedly decreased, and the protein level of P62 was significantly increased in the model group; these changes were effectively mitigated by treatment with either resveratrol or aminophylline alone ( Figure 4B). Furthermore, treatment with selisistat significantly abrogated the changes in the levels of autophagy-related proteins caused by aminophylline in the CRF model rats ( Figure 4C,D).

**Figure 5:**
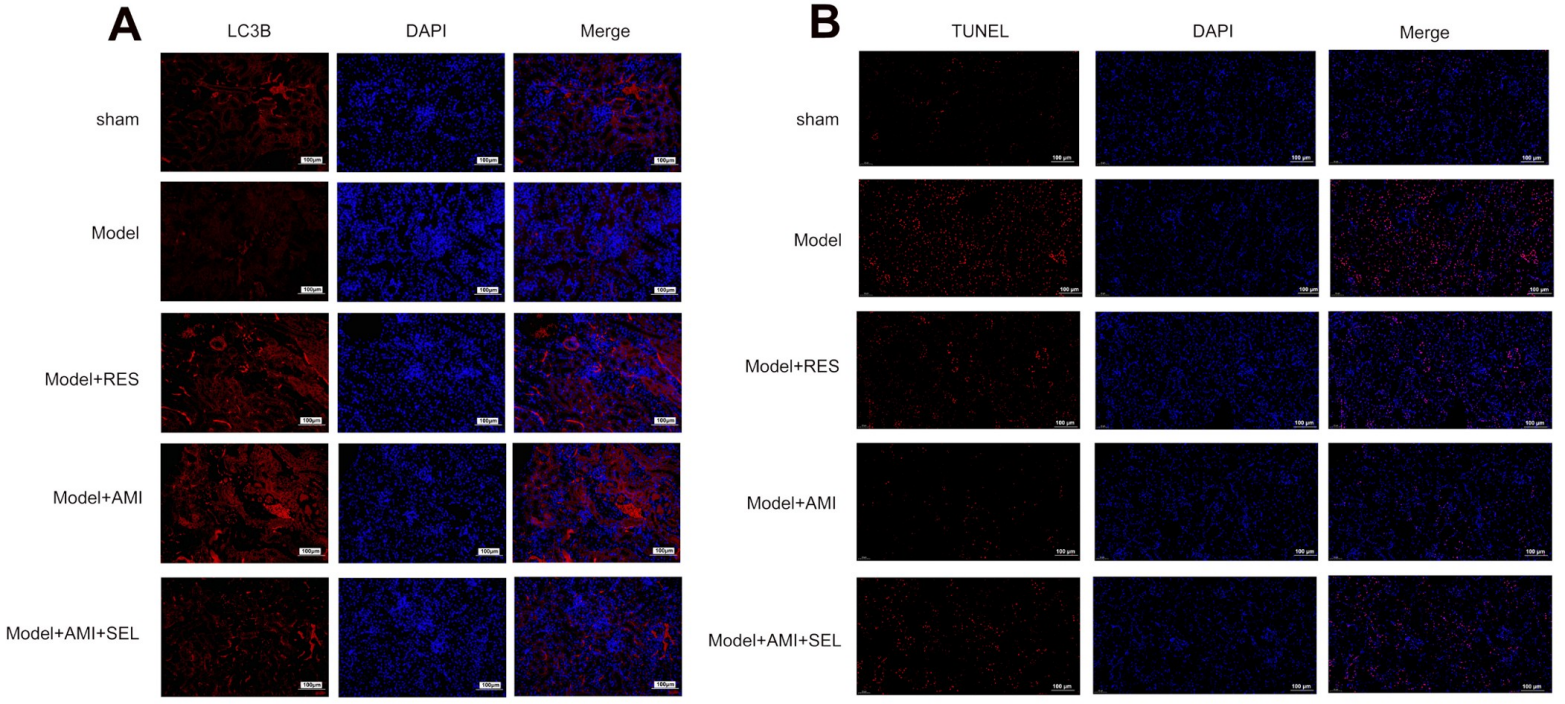
Figure 5 Changes in LC3B protein expression and cell apoptosis in aminophylline-treated CRF model rats after selisistat treatment (A) In vivo expression of the LC3B protein in CRF model rats after treatment with resveratrol or a combination of aminophylline and selisistat. LC3B expression in vivo was measured by immunofluorescence staining. (B) Regulation of renal cell apoptosis by resveratrol or the combination of aminophylline and selisistat in the rat CRF model. Kidney cell apoptosis was analyzed by TUNEL staining. AMI: aminophylline; RES: resveratrol; SEL: selisistat; LC3B: microtubule-associated protein 1 light chain 3B.

In addition, our data showed that the levels of cleaved poly (ADP-ribose) polymerase (PARP) and caspase 3 in the CRF model group were significantly greater than those in the sham group ( Figure 4E,F). However, treatment with either resveratrol or aminophylline alone significantly reduced the increases in cleaved PARP and caspase 3 protein levels in the CRF model rats. Additionally, the reductions in cleaved PARP and caspase 3 protein levels in the aminophylline-treated CRF model rats were partially mitigated by combined treatment with selisistat. Treatment of CRF model rats with resveratrol produced changes in LC3B protein levels that were similar to those produced by aminophylline treatment, and selisistat treatment greatly mitigated the changes in LC3B protein levels caused by aminophylline treatment ( Figure 5A). TUNEL staining showed that renal cell apoptosis in the CRF model rats was greatly suppressed by either resveratrol or aminophylline treatment alone ( Figure 5B). Selisistat treatment partially restored glomerular cell apoptosis in CRF model rats treated with aminophylline alone. These results proved that the modulation of renal cell autophagy and apoptosis by aminophylline in the CRF model rats was mediated by the activation of SIRT1.

### Aminophylline repressed renal damage and apoptosis in CRF model rats by regulating autophagy

**Figure 6:**
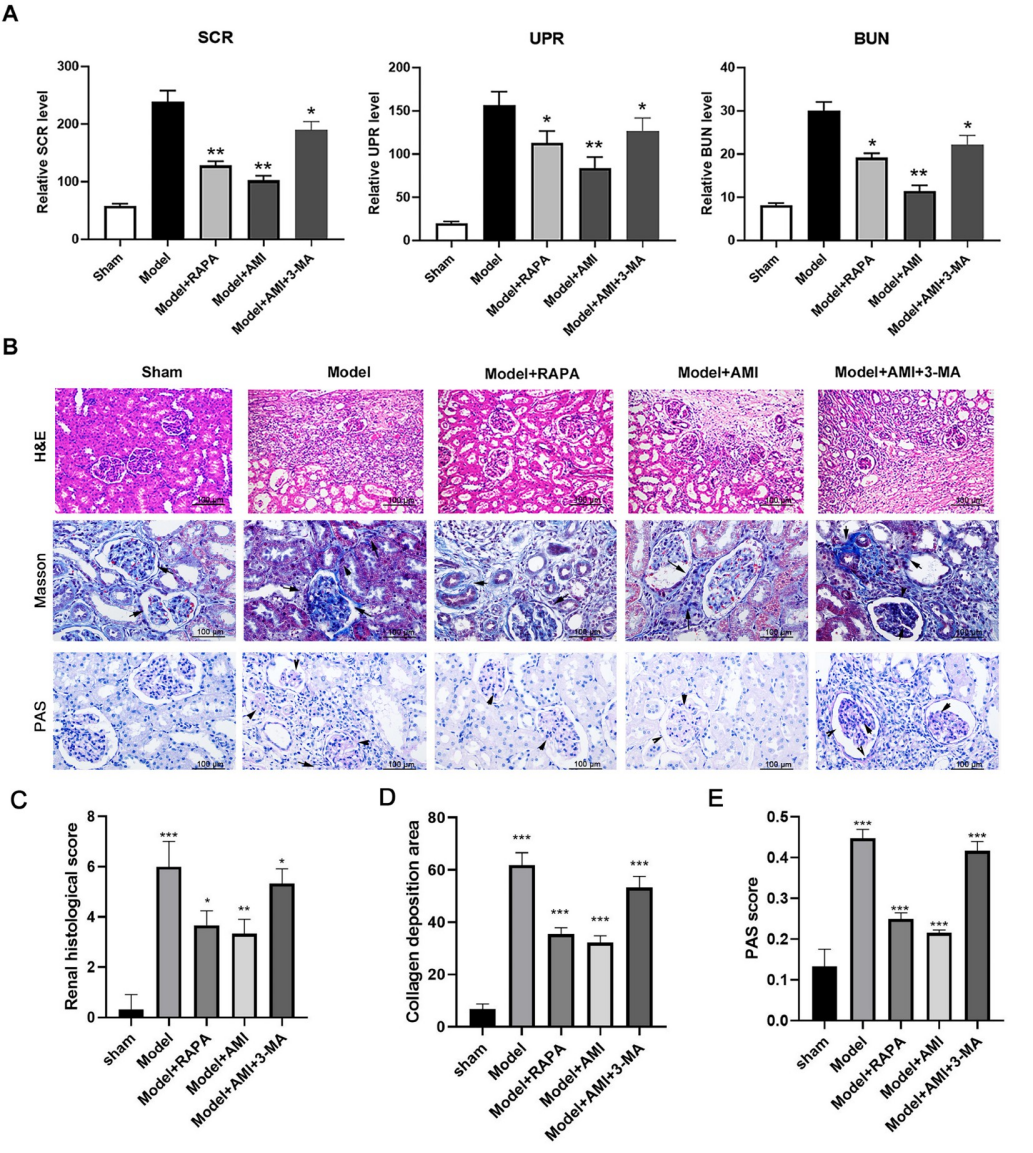
Figure 6 Involvement of autophagy in aminophylline-regulated CRF development (A) The levels of SCR, UPR, and BUN in CRF model rats treated with RAPA or a combination of aminophylline and 3-MA. The levels of SCR, UPR, and BUN were detected by ELISA. (B) Histological changes in the renal tissues of CRF model rats treated with RAPA or a combination of aminophylline and 3-MA. Tissue injury, interstitial fibrosis, and the accumulation of extracellular matrix in rat kidneys were analyzed by H&E staining, Masson staining, and PAS staining, respectively. (C) Renal histology scores determined by H&E staining. (D) Collagen deposition as detected by Masson staining. (E) PAS scores. The black arrows indicate areas of collagen deposition (Masson staining) and areas of glycogen deposition (PAS staining). RAPA: rapamycin; 3-MA: 3-methyladenine; SCR: serum creatinine; UPR: urinary protein; BUN: blood urea nitrogen. * P<0.05, ** P<0.01, *** P<0.001 vs sham.

To assess the mediating roles of autophagy, we treated CRF model rats with an autophagy inducer (RAPA, rapamycin) and an autophagy inhibitor (3-MA, 3-methyladenine). We found that RAPA treatment downregulated the levels of SCR, UPR, and BUN in the model group, and treatment with 3-MA partially restored the levels of SCR, UPR, and BUN in the model group treated with aminophylline ( Figure 6A). H&E staining revealed that RAPA treatment significantly alleviated renal tissue damage, interstitial fibrosis, and extracellular matrix deposition in CRF model rats, similar to the effects of aminophylline treatment alone ( Figure 6B,C). Moreover, treatment with 3-MA greatly mitigated the aminophylline-induced inhibition of renal tissue damage, interstitial fibrosis, and extracellular matrix deposition in the CRF model rats ( Figure 6B,D). PAS staining also showed that both aminophylline and RAPA could reduce glycogen deposition in glomerular lesions and reduce the symptoms of basement membrane thickening. However, when 3-MA treatment occurred at the same time as aminophylline intervention, the efficacy of aminophylline was inhibited. PAS staining also verified that the therapeutic effects of aminophylline is resulted from its activation of autophagy ( Figure 6B,E).

**Figure 7:**
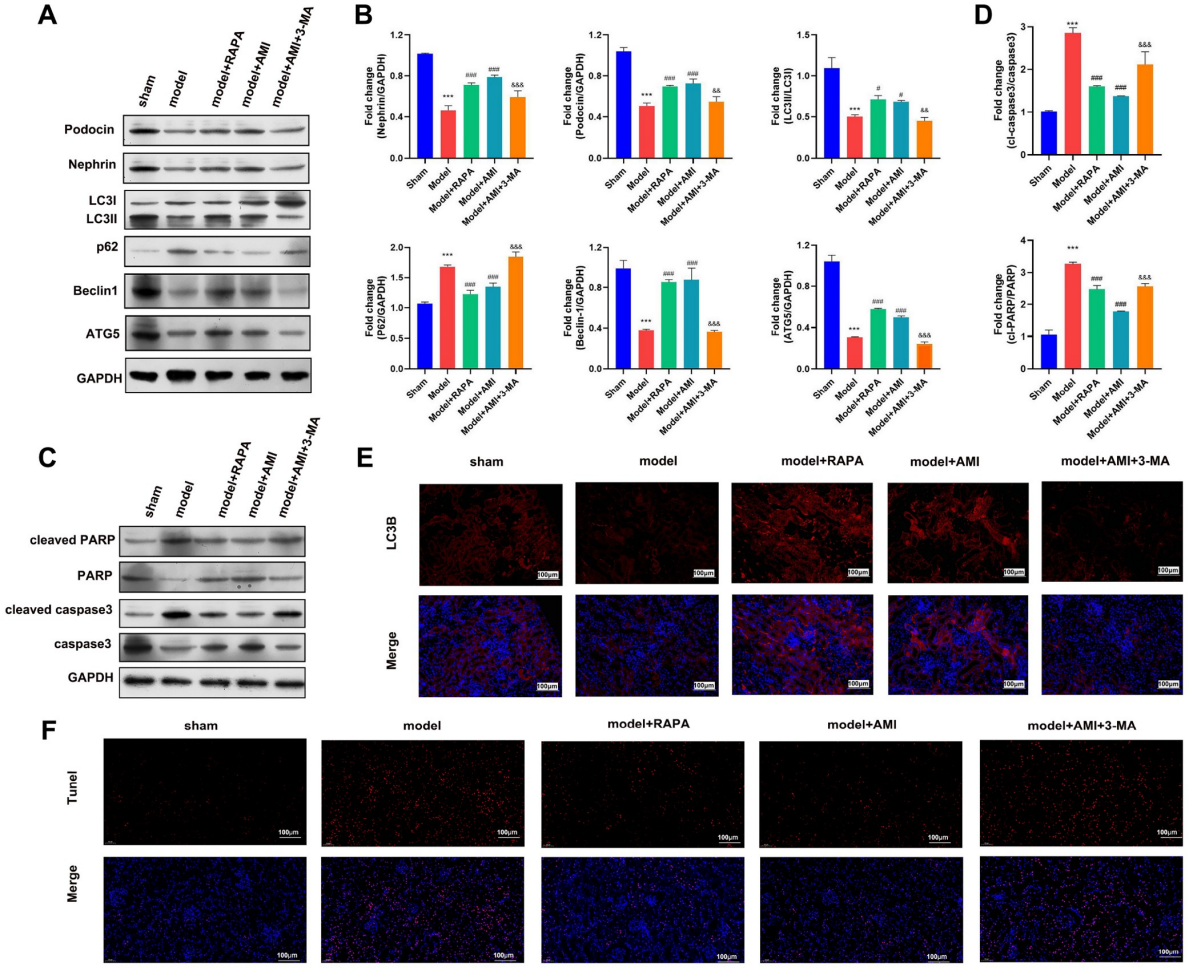
Figure 7 Aminophylline regulated apoptosis in CRF model rats by modulating autophagy (A) Changes in autophagy- and apoptosis-related marker proteins in CRF model rats treated with RAPA or a combination of aminophylline and 3-MA. (B) Quantitative analysis of LC3B, Beclin1, p62, and atf5 protein expressions. (C) Western blot analysis was performed to detect relative levels of protein expression, and GAPDH served as an internal standard. (D) Quantitative analysis of PARP and caspase3 protein expressions. (E) Levels of the LC3B protein in CRF model rats treated with resveratrol or a combination of aminophylline and selisistat. In vivo LC3B expression was detected by immunofluorescence staining. (F) Renal cell apoptosis in CRF model rats after treatment with RAPA or a combination of aminophylline and 3-MA. Rat renal cell apoptosis was analyzed by TUNEL staining. RAPA: rapamycin; 3-MA: 3-methyladenine. *** P<0.001 vs the sham group; # P<0.05, ### P<0.001 vs the model group; && P<0.01, &&& P<0.001 vs the model+AMI group.

In addition, RAPA treatment increased the protein levels of nephrin, podocin, Beclin-1, LC-3B, and ATG5 and downregulated the protein level of P62 in the model group, while 3-MA treatment effectively mitigated the aminophylline-induced changes in the protein levels in the model group ( Figure 7A,B). Similarly, the cleavage of PARP and caspase-3 in the model group was also greatly inhibited by RAPA treatment, and 3-MA treatment partially restored the cleavage of PARP and caspase-3 in the model group treated with aminophylline ( Figure 7C,D). We also found that the level of the LC-3B protein in the CRF model rats was greatly increased by treatment with either RAPA or aminophylline alone, while 3-MA treatment greatly repressed the increase in the LC-3B protein level caused by aminophylline in the CRF model rats ( Figure 7E). Consistent with these findings, treatment with either RAPA or aminophylline effectively suppressed renal cell apoptosis in the CRF model rats, and treatment with 3-MA partially reversed renal cell apoptosis in the CRF model rats treated with aminophylline ( Figure 7F). These results confirmed that aminophylline alleviated renal tissue damage and suppressed renal cell apoptosis via its modulation of autophagy.

## Discussion

The clinical management of CRF is still greatly limited by the poor availability or affordability of current treatments, and the development of new therapeutic molecules is considered to be a promising solution to this problem [18]. As a potent regulator of the second messengers cAMP and cGMP, aminophylline has been used or tested for the treatment of various human disorders, including acute kidney injury [23]. However, its functions in regulating CRF development and progression, as well as its possible cellular and molecular mechanisms, remain unknown. In the present study, we demonstrated for the first time that aminophylline could effectively inhibit renal tissue damage and cell apoptosis in CRF model rats. Moreover, the inhibitory effects of aminophylline on CRF progression were also shown to be mediated by its modulation of the autophagy process. In terms of molecular mechanisms, we discovered the essential roles played by the SIRT1/AMPK/mTOR signaling pathway in aminophylline-regulated autophagy and CRF progression. More importantly, our study showed that combined treatment with aminophylline and a SIRT1 agonist could effectively modulate SIRT1 signaling in a rat CRF model established by 5/6 nephrectomy. The results from our study help to verify the therapeutic effects of aminophylline, especially in treating renal disorders. In addition, they also provide a basis for its use in clinical applications. Our analyses further highlight the significance of autophagy and its regulation of signaling pathways as promising targets for CRF prevention and treatment.

The search for new drugs to treat CRF and CDKs has attracted great attention in the renal pharmacology research community in recent decades. CRF progression and its complications, such as vascular calcification, are associated with various cellular signaling pathways involving cGMP and cAMP [24]. Aminophylline can nonselectively inhibit phosphodiesterases that regulate cGMP and cAMP metabolism [25], suggesting its potential use in CRF treatment. On the other hand, the therapeutic effects of aminophylline on acute kidney injuries have been previously reported [26], and further studies suggested its use for regulating CRF development. In this study, we observed the effective alleviation of renal tissue dysfunction, interstitial fibrosis, and extracellular matrix deposition by aminophylline in a rat CRF model, which broadens our knowledge of the efficacy of aminophylline for treating renal disorders. Aminophylline has long been used as a classical therapeutic agent for treating pediatric diseases such as asthma in children, and the safety and efficacy of aminophylline have been convincingly validated by its long-term clinical use [27]. Moreover, a previous report also showed that aminophylline is capable of preventing contrast-induced nephropathy, which is one of the most common complications in CKD patients [28]. The potential regulation of other complications associated with CRF progression also deserves further investigation.

Autophagy has been widely implicated in the pathogenesis of renal disorders [29]. CRF development is accompanied by significant changes in autophagy, which also mediates the modulation of CRF progression via different mechanisms [30]. In this study, we showed that aminophylline effectively modulated autophagy in a rat CRF model, as shown by changes in the expressions of major autophagy marker proteins, such as Beclin-1, LC3B, P62, and ATG5. More importantly, we demonstrated the necessary mediating role of autophagy in aminophylline-regulated CRF progression by conducting combined treatment studies with an autophagy inhibitor (3-MA). Furthermore, the induction of autophagy is known to be regulated by SIRT1/AMPK/mTOR signaling cascades [31]. In this study, we also verified the possible role of the SIRT1/AMPK/mTOR signaling pathway in the aminophylline-induced modulation of autophagy and CRF development by conducting studies in which model rats were treated with the SIRT1 activator resveratrol and the SIRT1 inhibitor selisistat. These investigations provide evidence for the involvement of the SIRT1-mediated autophagy process in the suppression of CRF progression by aminophylline. Notably, considering the significance of autophagy in renal pathogenesis [32], the potential therapeutic roles of aminophylline in other kidney disorders also deserve exploration.

The interplay between autophagy and apoptosis is an essential cellular mechanism that drives the development of various human disorders [33]. Specifically, autophagy is known to modulate the initiation of apoptosis by regulating the activation of key apoptotic proteins. In this study, we demonstrated that aminophylline significantly suppressed renal cell apoptosis in CRF model rats, and this conclusion was supported by the detection of alterations in multiple apoptosis marker proteins, such as PARP and Caspase3. Furthermore, the regulation of renal cell apoptosis by autophagy during aminophylline-induced CRF suppression was further verified by resveratrol and 3-MA treatments, which revealed the crosstalk between autophagy and apoptosis underlying the therapeutic effects of aminophylline. On the other hand, the pathogenesis of CRF is mediated by an ongoing loss of nephrons [34], and dysregulation of renal cell apoptosis contributes to nephron loss during CRF [35]. The suppression of cell apoptosis by aminophylline also suggests its potential application in modulating nephrogenesis and regeneration for CRF treatment.

A major limitation of this study is that the involvement of the SIRT/AMPK/mTOR signaling pathway in the therapeutic effects of aminophylline on CRF was only based on analyses conducted in an animal model. The roles of SIRT1 signaling and other pathways in aminophylline-regulated renal cell autophagy in the context of CRF, as well as the underlying molecular mechanisms, should be further explored in renal cell models.

In summary, this study revealed that aminophylline effectively prevented renal cell apoptosis and CRF progression in a rat model by activating SIRT1-mediated autophagy. Our results provide new insights into the pharmaceutical role of aminophylline in the treatment of renal disorders and suggest that aminophylline is a promising new drug candidate for the clinical management of CRF patients.
